# Supplementary material for: SKP2 attenuates autophagy through Beclin1-ubiquitination and its inhibition reduces MERS-Coronavirus infection
Source: Nat Commun. 2019 Dec 18;10:5770. doi: 10.1038/s41467-019-13659-4 (PMC6920372; doi:10.1038/s41467-019-13659-4)
Supplement: Supplementary file 1 — Supplementary Information [file 41467_2019_13659_MOESM1_ESM.pdf]

Supplementary Information

To: SKP2 attenuates autophagy through Beclin1-ubiquitination and its inhibition reduces MERS-Coronavirus infection

By Gassen et al.

Supplementary Figures 1-10

Supplementary Tables 1-3

Supplementary References

Supplementary Figures

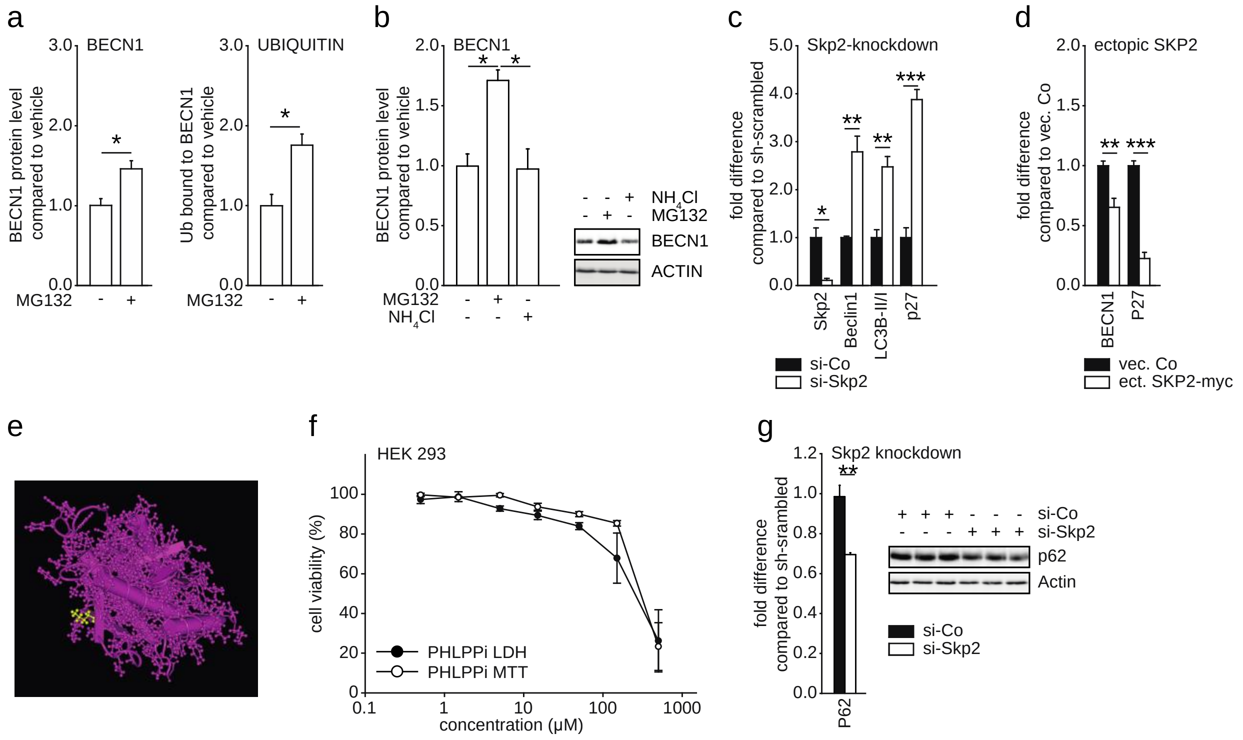

Supplementary Figure 1. *Supplements to main Figures 1-3.* **a** Quantification referring to main Figure 1b. **b** BECN1 is subject to proteasomal degradation. HEK293 cells were transfected with Ubiquitin-HA expressing plasmid and treated with the proteasome inhibitor MG132 (10 µM, 2 h) in combination with NH_4Cl (10 mM) as indicated. BECN1 was immunoprecipitated from whole cell extracts and probed for ubiquitination by western blotting[1]. **c** Quantification referring to main Figure 2g. **d** Quantification referring to main Figure 2h. **e** Lysine 402 is situated at an accessible site on the surface of BECN1. Depicted is the crystal structure of the evolutionary conserved domain of BECN1 (dbd # 4DDP_A)[2] with K402 highlighted in yellow. **f** Toxicity assays evaluating the PHLPP inhibitor (PHLPPi; NSC117079). VeroB4 cells were treated with the inhibitor (used in Figure 4d-h) at increasing concentrations as indicated for 1 h and cell viability was determined by the LDH (lactate dehydrogenase) and the MTT (tetrazole 3-(4,5-dimethylthiazol-2-yl-)-2,5-diphenyltetrazolium bromide) assay as described previously[3]. **g** Western blot and quantification of P62 upon downregulation of SKP2 by siRNA in HEK293 cells. In all panels, error bars denote the standard error of the mean, derived from n=3 (a-c,g) or n=6 (d) biologically independent experiments. * $p<0.05$, ** $p<0.01$, *** $p<0.001$ (b, 1 way ANOVA; a,c,d,g, t-tests; details in Supplementary Tables 1 and 2). Source data are provided as a Source Data file.

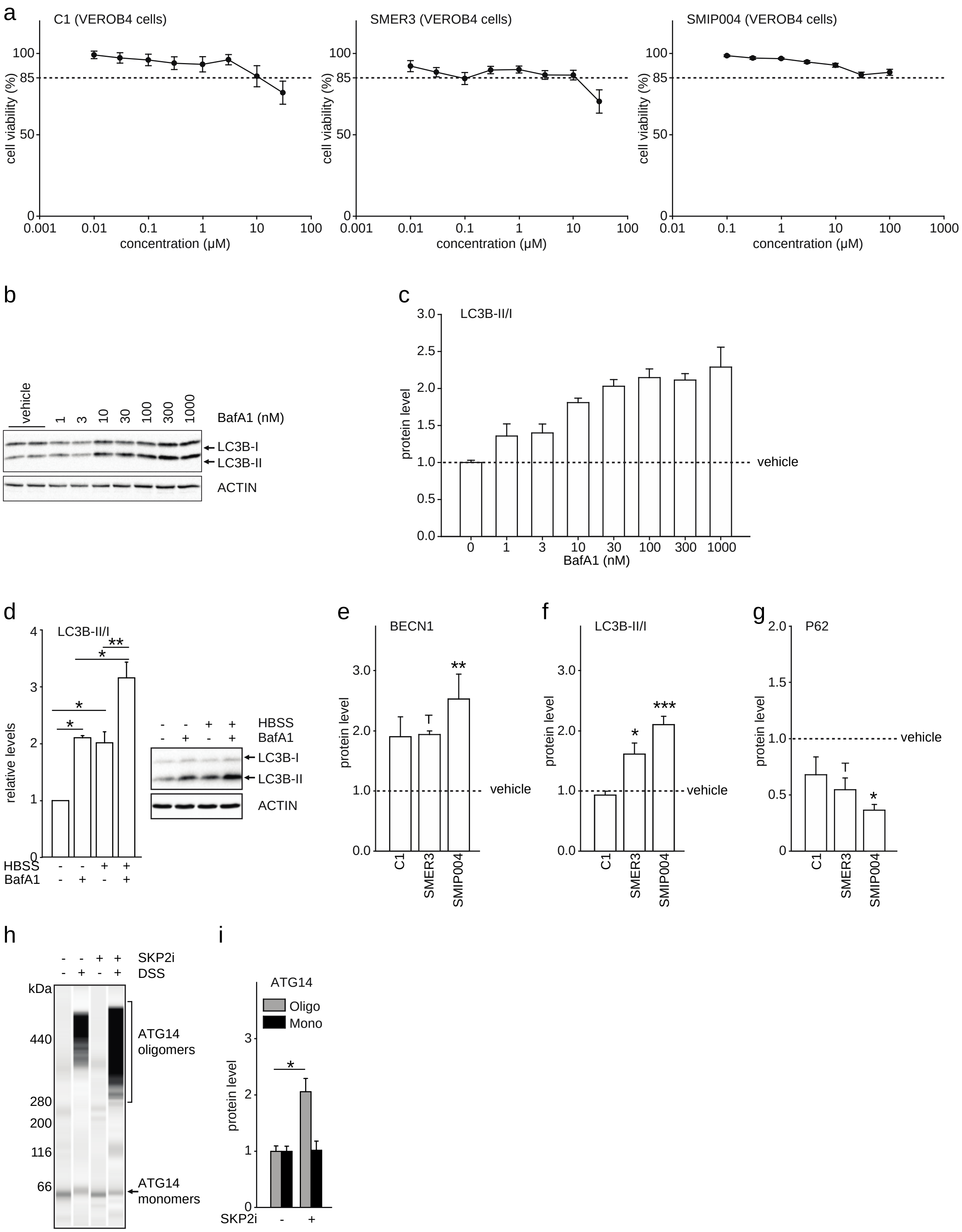

Supplementary Figure 2. See next page for the legend.

Supplementary Figure 2. *Comparison of SKP2 inhibitors and BafA1 titration* **a** Toxicity assays of SKP2 inhibitors. VeroB4 cells were treated with the SKP2 inhibitors C1, SMER3 and SMIP004 at increasing concentrations as indicated for 24 h and cell viability was determined by the MTT (tetrazole 3-(4,5-dimethylthiazol-2-yl-)-2,5-diphenyltetrazolium bromide) assay as described previously[3]. Based on these results, the concentrations used in the experiments of the main figures were chosen (i.e. at least 85% cell viability). **b,c** Titration of the BafA1 effect on LC3B lipidation in VeroB4 cells as required for the flux assays[1]. VeroB4 cells were exposed to increasing concentrations of BafA1 for 2 h before cells were lysed. The ratios of LC3B-II/I were determined by western blotting (a representative blot is shown). The graph represents the average levels + SEM of three independent experiments. Based on these data, 100 nM BafA1 was chosen for the experiments in all figures assessing autophagic flux as the concentration achieving complete block of autophagosome-lysosome fusion[1]. **d** HBSS control in VeroB4 cells to demonstrate the induction of autophagic flux. Representative Western blots are displayed. **e-g** VeroB4 cells were treated with the indicated inhibitors (C1 (3.3 µM), SMIP004 (10 µM), SMER3 (5 µM)) and the indicated protein levels were determined (mean + SEM of three independent experiments; quantification to the western blots in Figure 5e). **h,i** VeroB4 cells were exposed to SMIP004 for 24h, cross-linked with disuccinimidyl suberate (DSS, 75 µM) for 30 min and harvested. ATG14 homo-oligomerization was examined after western blotting (ProteinSimple). Quantification in (i). In all panels, error bars denote the standard error of the mean, derived from n=6 (a) or n=3 (c-g,i) biologically independent experiments. * $p<0.05$, ** $p<0.01$, *** $p<0.001$ (1 way ANOVA for b,e-g, 2 way ANOVA or d, and t-test for i, details in Supplementary Tables 1 and 2). Source data are provided as a Source Data file.

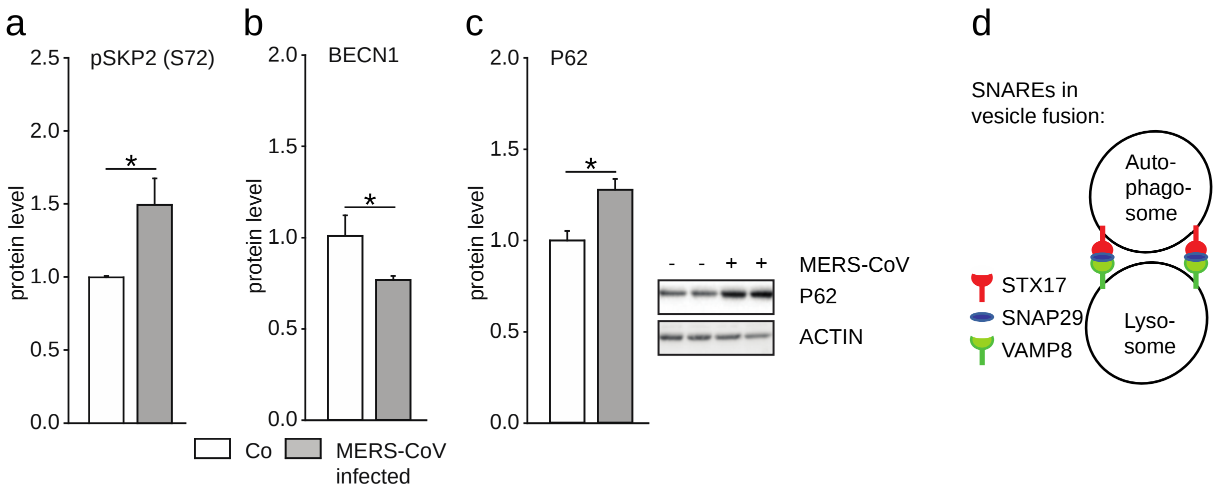

Supplementary Figure 3. *Effects of MERS-CoV on autophagy*. **a-c** VeroB4 cells were infected with MERS-CoV (MOI = 0.001) or left uninfected and harvested 48 h post infection (p.i.). Proteins were extracted for western blot analysis of markers of autophagy. Graphs represent the average protein levels (mock-treatment condition set to 1) + SEM of three independent experiments (corresponding representative western blot of BECN1 and pSKP2 in Figure 6a, of P62 integrated into panel c). Error bars denote the standard error of the mean, derived from n=4 (a, and controls of b,c) or n=8 (CoV of b,c) biologically independent experiments.p<0.05 (t-tests, details in Supplementary Table 2). **d** The scheme depicts the role of SNARE proteins in autophagy by pulling together autophagosome and lysosome, thus promoting autolysosome formation as the final step in autophagic degradation. It refers to the experiments shown in Figures 6h and 8f that analyse the association between the SNARE complex protein STX17 and VAMP8 or SNAP29. Source data are provided as a Source Data file.

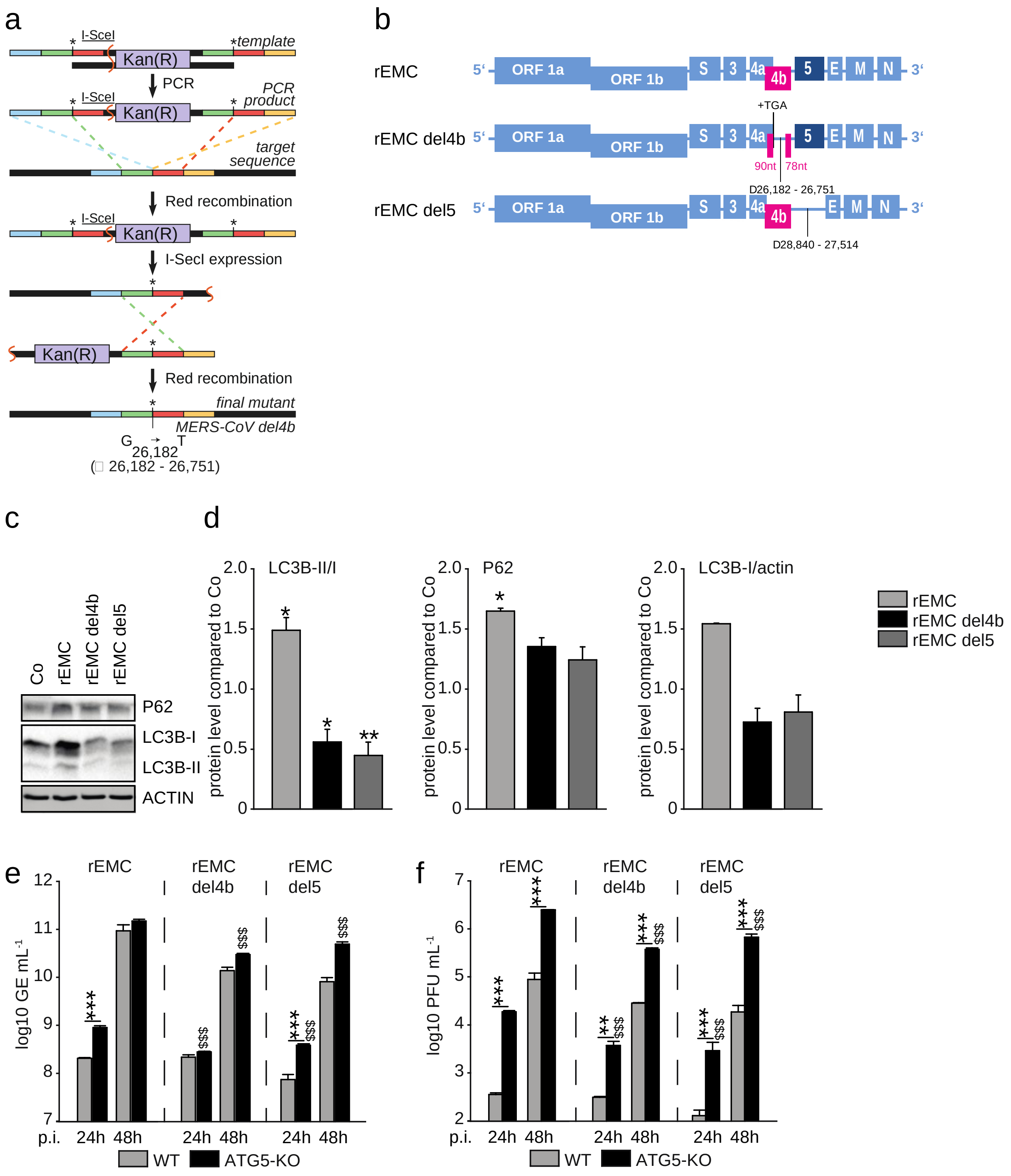

Supplementary Figure 4. *Analysis of p4b or p5-deleted MERS-CoV.* **a** Scheme of the recombinant MERS-CoV construction lacking orf4b or orf5. The generation of the recombinant MERS-CoV cDNA clone has been described[4]. The two step recombination procedure consists of the introduction of the mutated sequence alongside with the kanamycin selection marker and the

elimination of the kanamycin cassette using the unique I-SceI restriction site (detailed description in the Methods section). **b** Overview picture of the cloning strategy for construction the MERS-CoV mutations. For MERS-del4b, the nucleotide positions 26182-26751 were deleted. As shown in the picture, the complete orf4a is still present. For the MERS-del5 mutant, nucleotide positions 26840-27515 were deleted. Therefore, the ORF5 (encompassing 675 nucleotides from start to stop codon) was completely deleted. The regulatory element of ORF5 (TRS-5) was retained to maintain an equal number of subgenomic mRNAs that are generated during transcription **c,d** VeroB4 cells were infected with WT or mutant recombinant MERS-CoV (rEMC) (MOI = 0.001) and the indicated proteins were detected at 48h p.i.. The quantification is in reference to control (mock-infection, set to 1). **e,f** Deletion of ATG5 in the host cells VeroB4 does not abolish the difference in replication between mutant and wt MERS-CoV. VeroB4 wt or ATG5 KO cells were infected with wt or mutant recombinant MERS-CoV (rEMC) (MOI = 0.001) and genome copies (e) as well as plaque forming units (f) (PFU) were determined at 24 and 48 h p.i.. Unprocessed data are presented. In all panels, error bars denote the standard error of the mean, derived from n=3 biologically independent experiments. *'s indicate significant differences for WT vs ATG5-KO; $'s indicate differences for rEMC vs rEMCdel4b or rEMCdel5. * p<0.05, ** p<0.01, ***/$$$ p<0.001 (d, 1 way ANOVA, e,f, 2 way ANOVA, details in Supplementary Table 1. Source data are provided as a Source Data file.

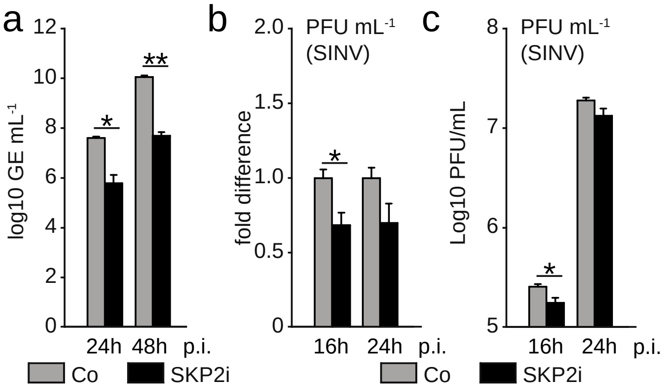

Supplementary Figure 5. *SKP2i restricts replication of MERS-CoV and, to a minor extent, Sindbis Virus.* **a** SKP2i limits MERS-CoV replication. VeroB4 wt cells were infected with MERS-CoV (MOI = 0.001) and genome copies were determined at 24 and 48 h p.i.. Unprocessed data are presented from which the fold differences presented in Figure 8A are derived. b,c VeroB4 wt cells were infected Sindbis Virus (SINV) (MOI = 0.0001), treated with 10 µM SKP2i (SMP004) and pfus were determined at 16 and 24 h p.i.. Data are presented as fold difference in comparison to DMSO vehicle control (co) treated cells (B), derived from the unprocessed data presented in (c). In all panels, error bars denote the standard error of the mean, derived from n=3 biologically independent experiments. * p<0.05, ** p<0.01 (t-tests, details in Supplementary Table 2. Source data are provided as a Source Data file.

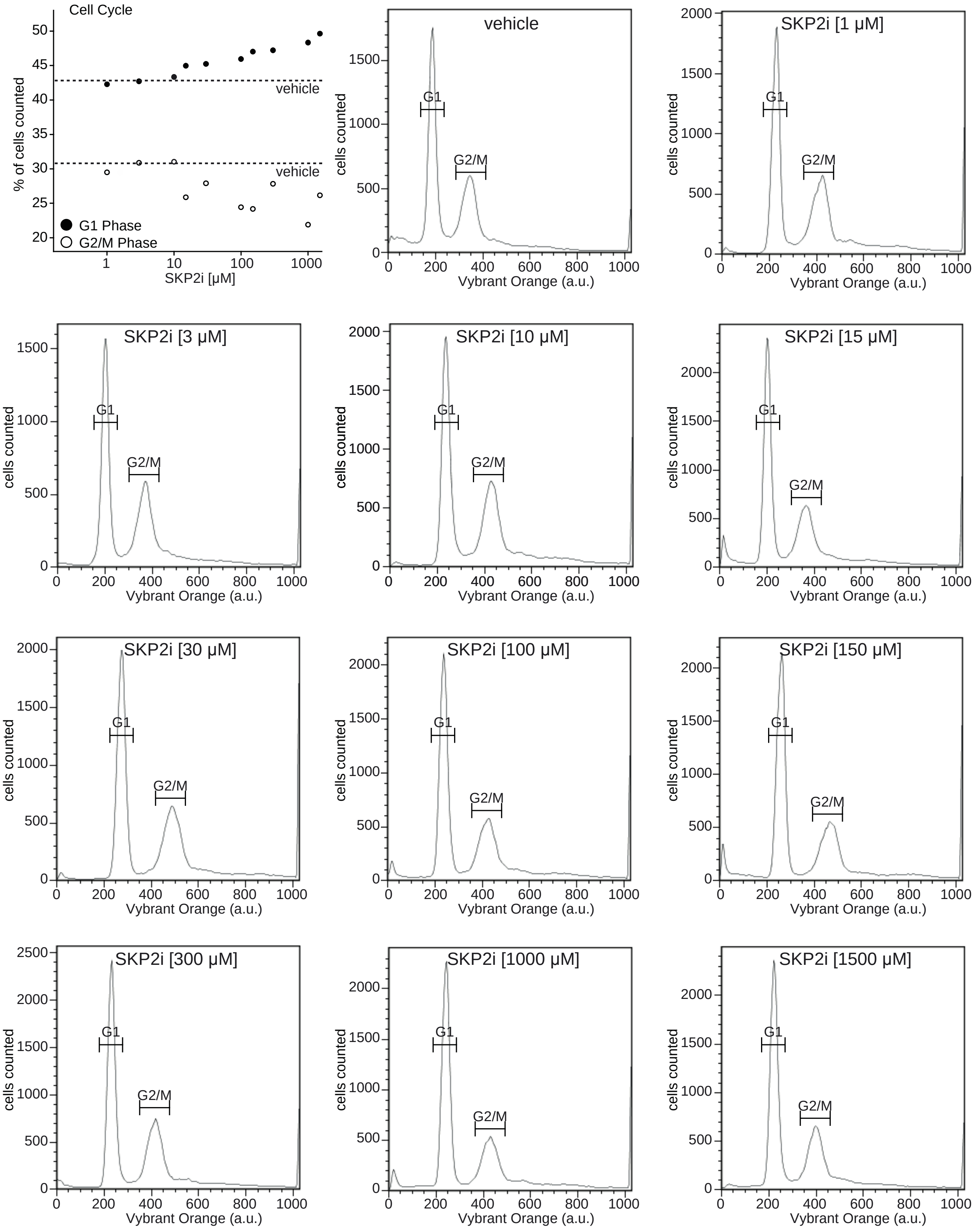

Supplementary Figure 6. See next page for the legend.

Supplementary Figure 6. *The SKP2-inhibitor (SKP2i) affects the cell cycle only at concentrations higher than 10 µM.* Vero4B cells were exposed to increasing concentrations of SKP2i (= SMIP004) as indicated. After 48 h, cells were stained with Vybrant Dyecycle orange and assessed for the cell cycle profile using FACS analysis. The first panel displays the summary of all the analyses showing cells in G1 (black circles) or G2/S (white circles) cell cycle phase; the dotted lines mark the respective percentages of vehicle-treated cells. All subsequent panels display the histograms recorded at the indicated concentrations of SKP2i. Source data are provided as a Source Data file.

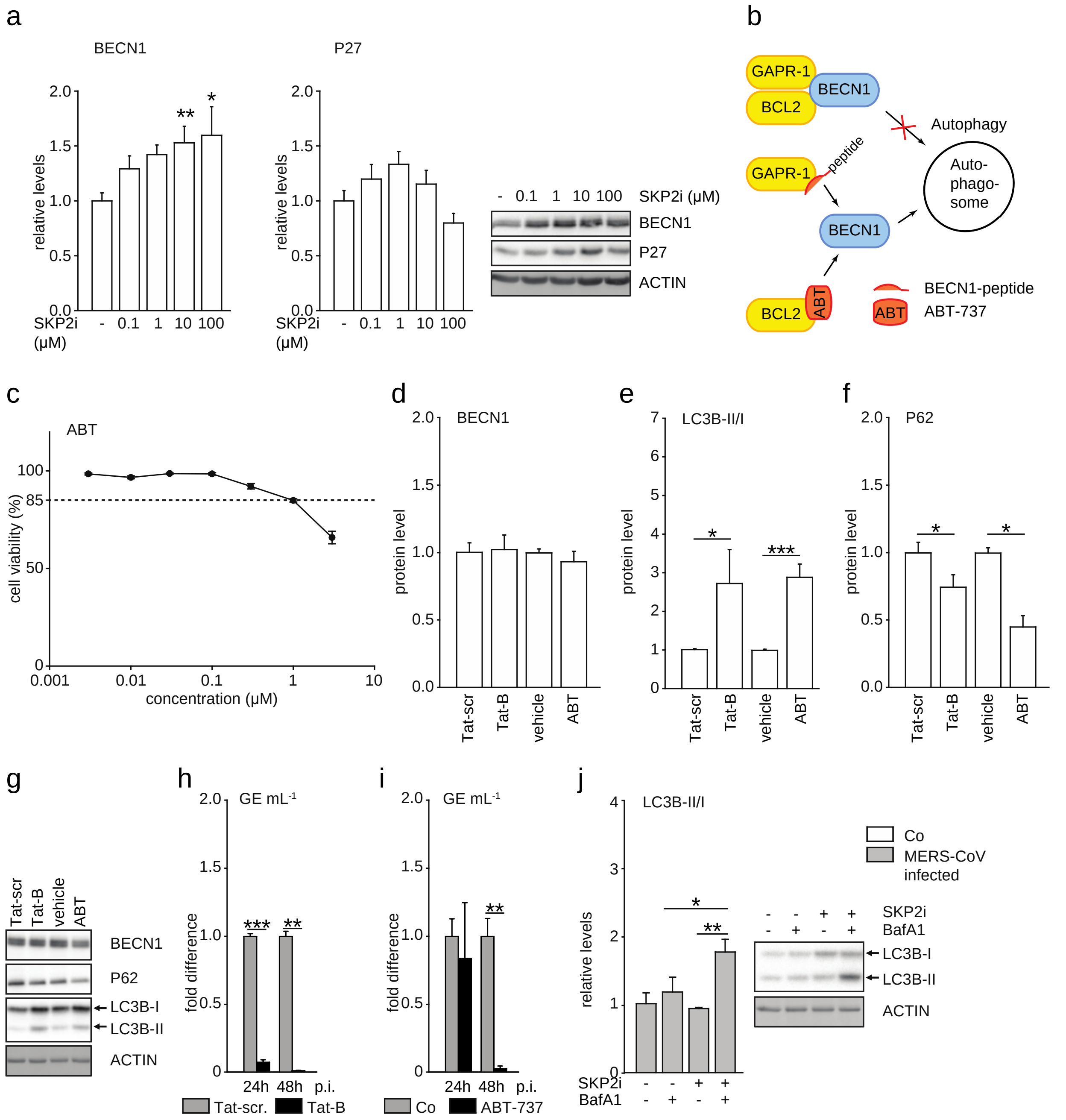

Supplementary Figure 7. *Effects of BECN1 targeting compounds on autophagy and MERS-CoV replication.* **a** VeroB4 cells were treated with SKP2i at the indicated concentrations for 48 h and the levels of BECN1 and P27 were determined by western blotting. **b** Scheme explaining the established effects of the BECN1-TAT peptide and the BH3-mimetic ABT-737[5, 6]. BECN1 is engaged in several protein interactions such as GAPR-1 or BCL2 which limits its function in

autophagy. The BECN1-Tat peptide blocks the interaction with GAPR-1, while ABT-737 blocks the interaction with BCL2. Thus, the compounds enhance the function of BECN1 in autophagy without changing its protein levels. **c** VeroB4 cells were treated with ABT-737 for 48 h and cell viability was determined by the MTT assay. **d-i** Effects of BECN1-targeting compounds. Non-infected (d-g) or MERS-CoV (MOI = 0.001)-infected VeroB4 cells (h,i) were cultivated in the presence of a BECN1-derived peptide (fused to the Tat transduction sequence, “Tat-B”) or ABT-737. Treatment with a scrambled version of Tat-B (Tat-scr.) or the vehicle of ABT-737 served as controls. After 48 h, the levels of BECN1, LC3B-II/I and P62 were determined by western blotting (d-g; control levels were set to 1), and virus genome copy numbers were determined by RT-PCR (fold difference refers to infected cells treated with scrambled peptide in h and to vehicle treatment in i). **j** SKP2i restores autophagic flux in MERS-CoV infected cells. VeroB4 cells were infected with MERS-CoV (MOI = 0.001), treated with SKP2i for 48 h, and incubated with bafilomycin A1 (BafA1, 0.1 µM) for 2 h before samples were taken at 48 h p.i.. The ratios of LC3B-II/I were determined by western blotting and quantified. In all panels, error bars denote the standard error of the mean, derived from n=12 (d-e control vehicle), n= 6 (d-e scrambled BECN1-TAT), n= 3 (d-e ABT737), n=3 (a), n=6 (c), n=2 (h and DMSO in i), n=3 (ABT737 in i) and n=4 (j) biologically independent experiments. * $p<0.05$, ** $p<0.01$, *** $p<0.001$ (2 way ANOVA in j, t-tests in d-f,h,i, details in Supplementary Tables 1 and 2). Source data are provided as a Source Data file.

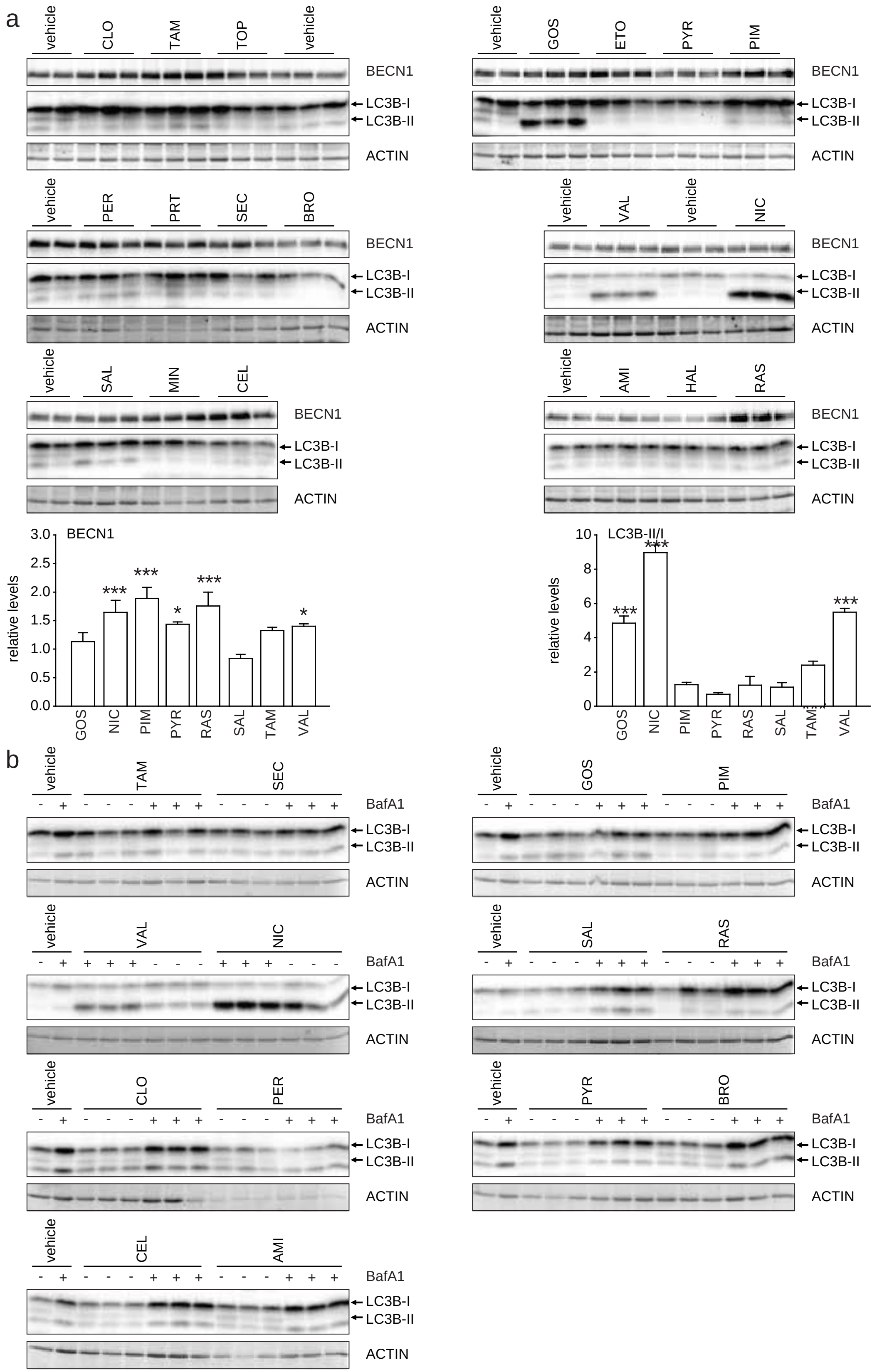

Supplementary Figure 8. See next page for the legend.

Supplementary Figure 8. *Effect of FDA-approved drugs on BECN1, LC3B-II/I and on autophagic flux*. **a** VeroB4 cells were treated with various FDA-approved drugs that were hypothesized to act on SKP2 (CLO, clomipramine, 10 µM; TAM, tamoxifen, 10 µM; TOP, topotecan, 0.01 µM; GOS, gossypol, 1 µM; ETO, etoposide, 10 µM; PYR, pyrvinium pamoate, 0.1 µM; PIM, pimozide, 0.3 µM; PER, perhexiline, 3 µM; PRT, parthenolide, 10 µM; SEC, securinine, 10 µM; BRO, bromocriptine, 10 µM; VAL, valinomycin, 5 µM; NIC, niclosamide, 10 µM; SAL, salinomycin, 0.3 µM; MIN, minocycline, 3 µM; CEL, celecoxib, 10 µM; AMI, amiloride, 10 µM; HAL, haloperidol, 30 µM; RAS, rasagiline, 1 µM). The levels of BECN1 and of LC3B-II/I were determined by western blotting after 48 h. **b** VeroB4 cells were treated with various FDA-approved drugs as in (a) and co-treatment with bafilomycin A1 (0.1 µM) was performed to evaluate the autophagic flux. Representative western blots are displayed in both panels. Quantification in Supplementary Table 3. In all panels, error bars denote the standard error of the mean, derived from n=12 (DMSO) or n=3 (each drug) biologically independent experiments. * $p<0.05$, *** $p<0.001$ (1 way ANOVA, details in Supplementary Table 1). Source data are provided as a Source Data file.

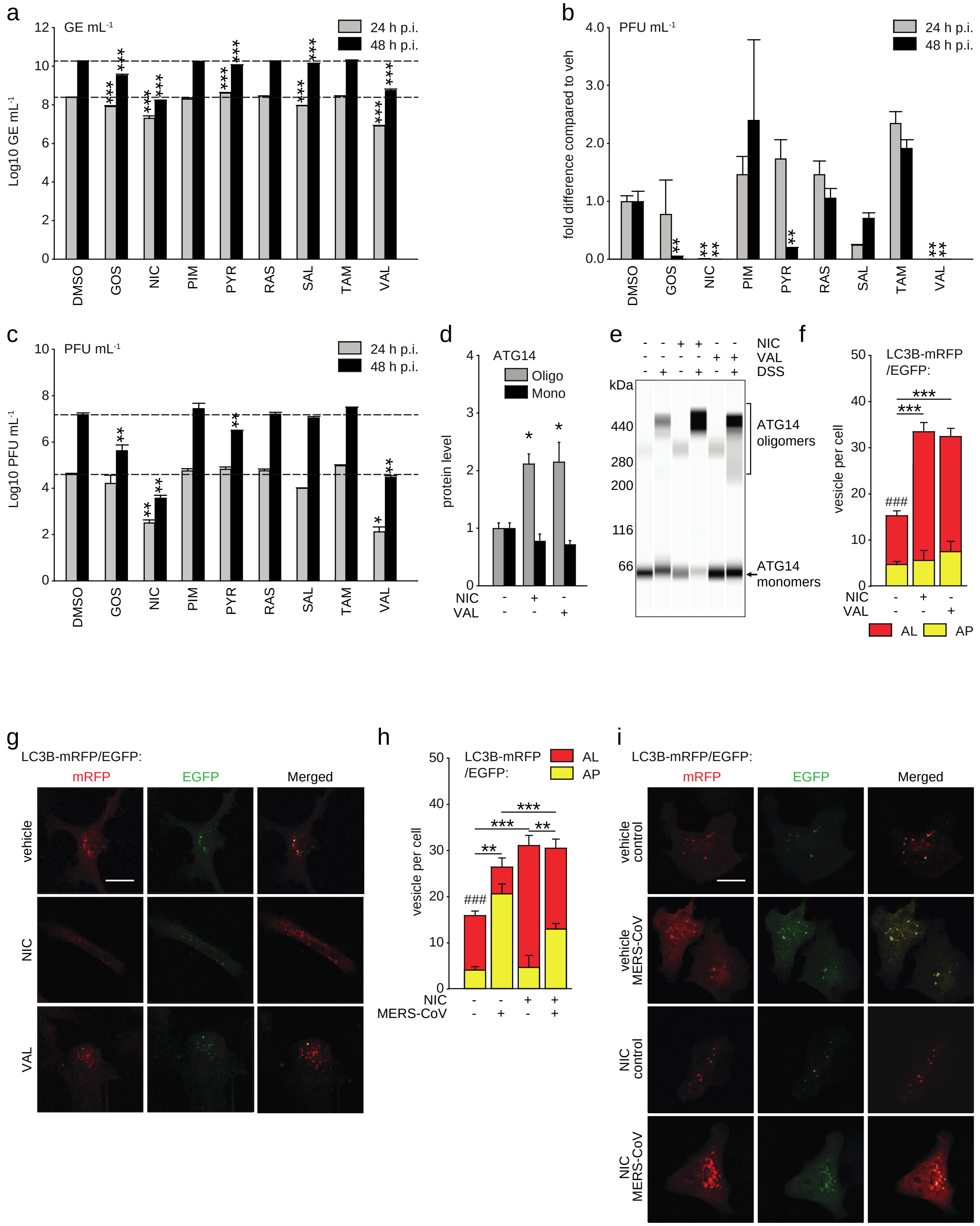

Supplementary Figure 9. See next page for the legend.

Supplementary Figure 9. *Effects of various drugs on viral replication and autophagic flux.* **a-c** VeroB4 cells were infected with MERS-CoV (MOI = 0.001), treated with the indicated drugs, and MERS-CoV genome copies (a) as well as PFUs (b,c) were determined 24 h and 48 h p.i.; data represent one of two independent experiments showing the mean values of biological triplicates and are displayed as log10 GE mL^-1 (a) (fold difference in Figure 9b) and fold difference of the pfus (b) derived from the data presented in panel c. **d,e** Niclosamide (NIC) and valinomycin (VAL) exert similar effects to SKPi on ATG14 oligomerization and autophagic flux. Oligomerization of ATG14 was determined after treatment of VeroB4 cells with NIC, VAL or vehicle for 48 h. A representative virtual blot (Protein Simple) is displayed (e). **f,g** VeroB4 cells were transfected with tandem fluorescent-tagged LC3B (mRFP that resists inactivation in autolysosomes and EGFP that is inactivated there), and treated with NIC, VAL or vehicle. The numbers of vesicles with both green and red fluorescence (autophagosomes, AP) and with red fluorescence only (autolysosomes, AL) were counted. Panel g, representative images; scale bar 25 µm. **h,i** VeroB4 cells were transfected with tandem fluorescent-tagged LC3, infected with MERS-CoV (MOI = 0.001) and treated with NIC or vehicle. 24 h post infection, cells were fixed and analyzed for fluorescence. The numbers of APs and ALs were counted as in f,g. Panel i, representative images; scale bar 25 µm. In all panels, error bars denote the standard error of the mean derived from n=3 biologically independent experiments for a-d and n=12 (NIC in f; MERS-untreated, MERS+NIC, MERS untreated in h), n=13 (vehicle in f, no virus+vehicle in h) or n=17 (CoV) different cells. * $p<0.05$; ** $p<0.01$, $^{***,\#\#\#}$ $p<0.001$ (1 way ANOVA for panels a-d,f; 2 way ANOVA for panel h, details in Supplementary Table 1). **,*** in panels f,h refer to the difference between the number of autolysosomes, $^{\#\#\#}$ to the difference between the total number of fluorescing vesicles. Source data are provided as a Source Data file.

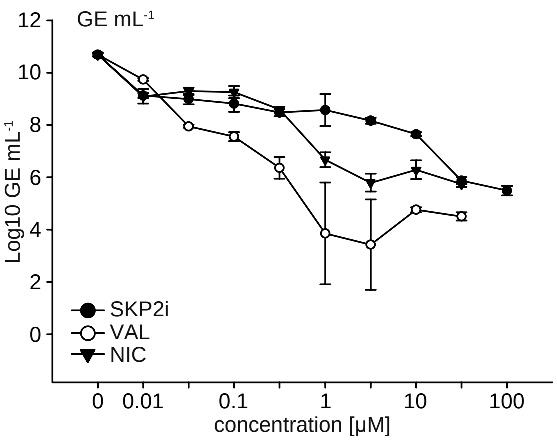

Supplementary Figure 10. *Concentration-dependent effects of SKP2i, niclosamide and valinomycin on MERS-CoV replication.* VeroB4 cells were infected with MERS-CoV (MOI = 0.001) and treated with drug. MERS-CoV genome copies were determined by real-time RT-PCR at 48 h p.i., data presented as GE on a log10-y-axis. and form the basis for the calculation of relative virus growth presented in Figure 9c. Statistical quantification and details in supplementary Table 1. Source data are provided as a Source Data file.

Supplementary Tables

Supplementary Table 1

Main Fig	parameter	ANOVA	variable 1		variable 2		var. 1 * var. 2	
1a	BECN1 (mRNA)	1 Way, variable 1 = overexpression	F=0.104	p=0.902				
	BECN1 (prot.)		F=15.103	p=0.005				
1d	BECN1	2 Way, 1 treatment x 2 CHX t	F=48.290	p<0.001	F=77.123	p<0.001	F=15.673	p<0.001
1f		2 Way, 1 treatment x 2 chase t	F=30.167	p<0.001	F=68.915	p<0.001	F=7.384	p<0.001
2d		2 Way, 1 condition x 2 CHX t	F=46.123	p<0.001	F=131.196	p<0.001	F=12.304	p<0.001
2f		2 Way, 1 condition x 2 chase t	F=45.956	p<0.001	F=130.216	p<0.001	F=10.663	p<0.001
2k		2 Way, 1 siRNA x 2 CHX time	F=21.780	p<0.001	F=7.162	p=0.017	F=2.659	p=0.083
2m		2 Way, 1 siRNA x 2 chase t	F=19.132	p<0.001	F=7.251	p=0.015	F=1.204	p=0.340
4c		2 Way, 1 vector x 2 CHX time	F=4.596	p=0.009	F=123.099	p<0.001	F=1.030	p=0.439
4f		2 Way, 1 treatment x 2 chase t	F=6.443	p=0.015	F=23.767	p<0.001	F=0.771	p=0.517
4h		2 Way, 1 condition x 2 CHX t	F=22.424	p<0.001	F=82.377	p<0.001	F=3.831	p=0.002
4j		2 Way, 1 treatment x 2 CHX t	F=8.311	p=0.002	F=46.786	p<0.001	F=3.857	p=0.008
4l		2 Way, 1 condition x 2 chase t	F=17.910	p<0.001	F=58.662	p<0.001	F=5.191	p=0.002
4m	LLPs	2 Way, 1 siRNA x 2 3-MA	F=7.145	p=0.015	F=6.247	p=0.021	F=4.484	p=0.047
5a	BECN1	2 Way, 1 treatment x 2 CHX t	F=66.250	p<0.001	F=180.983	p<0.001	F=14.011	p<0.001
5b		2 Way, 1 treatment x 2 chase t	F=9.180	p<0.001	F=58.380	p<0.001	F=2.473	p=0.021
5d	LLPs	2 Way, 1 treatment x 2 3-MA	F=4.106	p=0.006	F=37.299	p<0.001	F=3.776	p=0.009
5f	LC3B-II/I	2 Way, 1 BafA1x 2 SKP2i	F=42.122	p<0.001	F=14.189	p=0.003	F=1.406	p=0.259
6g		2 Way, 1 BafA1 x 2 virus inf.	F=18.312	p<0.001	F=5.310	p=0.030	F=27.854	p<0.001
8c	tfLC3B-II/I, total	2 Way, variable 1 = treatment; variable 2 = MERS-CoV	F=84.868	p<0.001	F=149.483	p=0.030	F=43.000	p<0.001
	tfLC3B-II/I (APs)		F=9.948	p=0.003	F=12.335	p=0.001	F=4.706	p=0.035
9a	LC3B-II/I	2 Way, 1 BafA1x 2 treatment	F=80.693	p<0.001	F=202.331	p<0.001	F=17.675	p<0.001
9b/	Virus GE 24h	1 Way, variable 1 = treatment	F=79.438	p<0.001				
	Virus GE 48h		F=195.092	p<0.001				
9c	Virus growth	1 Way, variable 1 = SKP2i	F=8.79	p<0.0001				
		1 Way, variable 1 = VAL	F=14.77	p<0.0001				
		1 Way, variable 1 = NIC	F=14.6	p<0.0001				
		1 Way, variable 1 = VAL	F=275.1	p<0.0001				
		1 Way, variable 1 = NIC	F=108.7	p<0.0001				

Details of the ANOVA statistical analyses.

Supplementary Table 1 (continued)

Suppl. Fig.	parameter	ANOVA	variable 1		variable 2		var. 1 * var. 2	
9a	Virus GE 24h	1 Way, variable 1 = treatment	F=79.438	p<0.001				
	Virus GE 48h		F=195.092	p<0.001				
1b	BECN1	1 Way, variable 1 = treatment	F=11.604	p=0.009				
2d	LC3B-II/I	2 Way, 1 BafA1 x 2 HBSS	F=43.302	p<0.001	F=36.694	p<0.001	F=0.0102	p=0.922
2e	BECN1	1 Way, variable 1 = treatment	F=5.121	p=0.017				
2f	LC3B-II/I	1 Way, variable 1 = treatment	F=16.245	p<0.001				
2g	P62	1 Way, variable 1 = treatment	F=3.993	p=0.034				
4d	LC3B-II/I	1 Way, variable 1 = rEMC	F=26.059	p<0.001				
	P62		*H=9.596	p=0.022				
	LC3B-I/actin		*H=8.192	p=0.042				
4e	Virus GE 24h	2 Way, 1 host genotype x 2 virus genotype	F=42.481	p<0.001	F=109.296	p<0.001	F=34.757	p<0.001
	Virus GE 48h		F=27.043	p<0.001	F=8.679	p=0.012	F=1.367	p=0.292
4f	Virus pfu 24h	2 Way, 1 host genotype x. 2 virus genotype	F=110.515	p<0.001	F=343.454	p<0.001	F=106.731	p<0.001
	Virus pfu 48h		F=270.783	p<0.001	F=744.744	p<0.001	F=238.121	p<0.001
7j	LC3B-I/actin	2 Way, 1 BafA1 x 2 SKP2i	*H=8.192	p=0.042	F=2.877	p=0.116	F=2.258	p=0.159
8ab Tab. 3	BECN1	1 Way, variable 1 = treatment	F=11.239	p<0.001				
	LC3B-II/I		F=124.079	p<0.001				
9b	Virus pfu 24h	1 Way, variable 1 = treatment	*H=21.401	p=0.006				
	Virus pfu 48h		*H=24.123	p=0.002				
9c	Virus pfu 24h	1 Way, variable 1 = treatment	*H=21.252	p=0.007				
	Virus pfu 48h		*H=24.123	p=0.002				
9d	Atg14 (mono)	1 Way, variable 1 = treatment	F=2.119	p=0.201				
	Atg14 (oligo)		F=8.231	p=0.019				
9f	tfLC3B-II/I, total	1 Way, variable 1 = treatment	*H=24.494	p<0.001				
	tfLC3B-II/I (APs)		*H=0.679	p=0.712				
9h	tfLC3B-II/I, total	2 Way, 1 treatm. x 2 virus inf.	F=27.695	p<0.001	F=7.405	p=0.009	F=9.235	p=0.004
	tfLC3B-II/I (APs)		F=3.808	p=0.057	F=47.231	p<0.001	F=5.183	p=0.028
10	Virus growth	1 Way, variable 1 = SKP2i	F=70.11	p<0.0001				
		1 Way, variable 1 = VAL	F=275.1	p<0.0001				
		1 Way, variable 1 = NIC	F=108.7	p<0.0001				

Details of the ANOVA statistical analyses.

* Kruskal-Wallis 1 Way ANOVA on Ranks

Supplementary Table 2

Fig.	parameter	interaction	t-value	p-value
6b	BECN1 K48-polyubiquitination	Mock x MERS-CoV	t= -3.117	p=0.021
6c	tfLC3B-II/I (total punctae)		t=-3.851	p<0.001
	tfLC3B-II/I (APs)		T=108.000	*p=0.010
6f	ATG14 (monomers)		t=-0.151	p=0.444
	ATG14 (oligomers)		t=-7.470	p<0.001
6h	VAMP8 binding to STX17		t=4.487	p=0.002
	SNAP29 binding to STX17		t=2.596	p=0.041
7a	PFU mL^-1, raw	WT x ATG5KO 24h	t= -40,736	p<0.001
		WT x ATG5KO 48h	t= -10,475	p<0.001
	PFU mL^-1, fold difference	WT x ATG5KO 24h	t=-16,368	p<0.001
		WT x ATG5KO 48h	t=-55,193	p<0.001
7b	GE mL^-1, raw	WT x ATG5KO 24h	t= -15,506	p<0.001
		WT x ATG5KO 48h	t= -1,803	p=0.146
	GE mL^-1, fold difference	WT x ATG5KO 24h	t=-7,865	p=0.001
		WT x ATG5KO 48h	t=-1,706	p=0.163
8a	GE mL^-1, fold difference	vehicle x SKP2i, 24h	t=3,629	p=0.0222
		vehicle x SKP2i, 48h	t=4,109	p= 0.0147
8e	Atg14 (monomers)	vehicle x SKP2i	t=0.671	p=0.269
	Atg14 (oligomers)		t=-5.181	p=0.003
8f	VAMP8 binding to STX17		t=-3.025	p=0.012
	SNAP29 binding to STX17		T=18.000	*p=1.000
S.1a	BECN1 (protein levels)	vehicle x MG132	t=-3.516	p=0.025
	Ub binding to BECN1		t=-3.820	p=0.019
S.1c	SKP2	si-Co x si-SKP2	t=4.294	p=0.013
	BECN1		t=-5.509	p=0.005
	P27		t=-9.791	p<0.001
	LC3BII/I		t=-5.477	p=0.005
S.1d	BECN1	vector x ect. SKP2-myc	t=4.062	p=0.002
	P27		t=11.827	p<0.001
S.1g	P62	si-Co x si-SKP2	t=4.952	p=0.008
S.2i	ATG14 monomers	vehicle x SKP2i	t=-0.106	p=0.921
	ATG14 oligomers		t=-4.194	p=0.014
S.3a	pSKP2^{S72}	Mock x MERS-CoV	T=10,000	p=0.029
S.3b	BECN1		T=40.000	*p=0.016
S.3c	P62		t=-3.037	p=0.006
S.5a	GE mL^-1, raw	vehicle x SKP2i, 24h	t=4.481	p=0.011
		vehicle x SKP2i, 48h	t=8.403	p= 0.0011
S.5b	PFU mL^-1, fold	vehicle x SKP2i, 16h	t=3.16	p=0.0342
		vehicle x SKP2i, 24h	t=2.026	p= 0.1128
S.5c	PFU mL^-1, raw	vehicle x SKP2i, 16h	t=2.795	p=0.0491
		vehicle x SKP2i, 24h	t=2.06	p= 0.1084
S.7d	BECN1	Tat-scr x Tat-B	t=-0.164	p=0.436
		vehicle x ABT	t=0.918	p=0.188
S.7e	LC3B-II/I	Tat-scr x Tat-B	T=21.000	p=0.002
		vehicle x ABT	T=42.000	*p=0.012
S.7h	GE mL^-1, fold difference	Tat-scr x Tat-B, 24h	t=32.88	p=0.0009
		Tat-scr x Tat-B, 48h	t=24.75	p= 0.0016
S.7i	GE mL^-1, fold difference	vehicle x ABT, 24h	t=0.3040	p=0.781
		vehicle x ABT, 48h	t=9.6730	p= 0.0023
S.7f	P62	Tat-scr x Tat-B	t=2.082	p=0.032
		vehicle x ABT	T=6.000	*p=0.012

Details of the t-test analyses. Supplementary Figures are referred to with the abbreviation S.;
* Mann-Whitney U statistic

Supplementary Table 3.

Drug[#]	Approved/tested Treatment	BECN1 change	LC3B Lipidation change	Effect on autophagic flux	MERS inhibition (log10, 24h/48h p.i.)
Amiloride (AMI)	Hypertension, Heart failure	0.80±0.06	1.19±0.05	1.10±0.05	ND
Bromocriptine (BRO)	Pituitary tumor, Parkinson's disease, type 2 diabetes	0.53±0.03	1.61±0.34	1.12±0.22	ND
Celecoxib (CEL)	Osteoarthritis, rheumatoid arthritis	1.73±0.22	0.90±0.18	1.07±0.07	ND
Clomipramine (CLO)	Depression	1.11±0.07	1.24±0.22	0.65±0.05	ND
Etoposide (ETO)	Cancer	1.44±0.20	0.86±0.14	ND	ND
Gossypol (GOS)	Cancer, Contraception	1.13±0.16	4.86±0.42 ***	1.57±0.22 ***	0.89±0.12 1.91±0.35
Haloperidol (HAL)	Psychosis	0.84±0.29	1.98±0.30	ND	ND
Minocycline (MIN)	Bacterial infection	1.88±0.31	0.43±0.05	ND	ND
Niclosamide (NIC)	Worm infection	1.65±0.21 ***	8.98±0.44 ***	1.66±0.13 ***	1.32±0.04 3.09±0.11
Parthenolide (PRT)	Various applications in herbal medicine	0.89±0.08	2.16±0.15	ND	ND
Perhexiline (PER)	Angina	1.26±0.30	0.53±0.02	ND	ND
Perphenazine (PEP)	Psychosis	0.97±0.06	2.34±0.71	1.36±0.29	ND
Pimozide (PIM)	Psychosis	1.89±0.20 ***	1.26±0.14	0.71±0.06	0.17±0.17 0.18±0.02
Pyrvinium pamoate (PYR)	Worm infection	1.44±0.04 *	0.71±0.09	0.95±0.05	0.13±0.06 2.22±0.09
Rasagiline (RAS)	Parkinson's disease	1.76±0.24 ***	1.24±0.51	1.09±0.14	0.19±0.01 0.05±0.00
Salinomycin (SAL)	Bacterial infection	0.84±0.06	1.12±0.27	1.55±0.14 **	1.40±0.18 0.36±0.07
Securinine (SEC)	Neurological related diseases	0.58±0.04	1.17±0.05	0.67±0.05	ND
Tamoxifen (TAM)	Breast cancer	1.33±0.06	2.41±0.23 ***	0.87±0.06	0.15±0.07 0.42±0.01
Topotecan (TOP)	Lung cancer, ovarian cancer	1.00±0.07	1.74±0.22	ND	ND
Valinomycin (VAL)		1.40±0.04 *	5.51±0.22 ***	1.84±0.08 ***	2.50±0.09 4.10±0.08

Clinically approved or tested drugs assessed for effects on autophagy and virus replication.

#All drugs are clinically approved, except for GOS, SAL and TOP which are clinically tested. The antibiotic valinomycin was included because it had shown antiviral effects on severe acute respiratory syndrome human coronavirus[7]. VeroB4 cells were infected with MERS-CoV (MOI = 0.001) and treated with various FDA-approved drugs that were hypothesized to act on SKP2. Concentrations of the drugs were 10 µM AMI, ETO, 10 µM, 1 µM GOS, 10 µM NIC; 0.3 µM PIM, 0.1 µM PYR, 1 µM RAS, 0.3 µM SAL, 10µM TAM, 5µM VAL, 10 µM BRO, 10 µM CEL, 10 µM CLO, 30 µM HAL, 3 µM MIN, 10 µM PRT, 3 µM PER, 1 µM PEP, 10 µM SEC, and 0.01 µM TOP. These concentrations were based on the results of the LDH and MTT toxicity assays and a threshold of 85% as minimal cell viability. The levels of BECN1 and of LC3B-II/I were determined by western blotting 48 h p.i.. Co-treatment with BafA1 (0.1 µM) was performed to evaluate the autophagic flux. Numbers for BECN1 change indicate the mean fold change ± SEM. LC3B lipidation represents the fold change ± SEM of the LC3B-II/I ratio by drug treatment in comparison to vehicle (DMSO) treatment. The drug effect on flux is revealed by the fold increase induced by BafA1 on the LC3B-II/I ratio (mean ± SEM, derived from n=3 biologically independent experiments). MERS-CoV genome copies were determined by RT-PCR 24 h and 48 h p.i.; data are presented as (log10) inhibition. * $p<0.05$, ** $p<0.01$, *** $p<0.001$ (1 way ANOVA, details in Supplementary Table 1).

Supplementary References

1. Klionsky, D. J. et al. Guidelines for the use and interpretation of assays for monitoring autophagy (3rd edition). *Autophagy* **12**, 1-222 (2016).

2. Huang, W. et al. Crystal structure and biochemical analyses reveal Beclin 1 as a novel membrane binding protein. *Cell Res* **22**, 473-489 (2012).

3. Zschocke, J. et al. Antidepressant drugs diversely affect autophagy pathways in astrocytes and neurons--dissociation from cholesterol homeostasis. *Neuropsychopharmacology* **36**, 1754-1768 (2011).

4. Muth, D. et al. Transgene expression in the genome of Middle East respiratory syndrome coronavirus based on a novel reverse genetics system utilizing Red-mediated recombination cloning. *J Gen. Virol. **98**, 2461-2469* (2017).

5. Shoji-Kawata, S. et al. Identification of a candidate therapeutic autophagy-inducing peptide. *Nature* **494**, 201-206 (2013).

6. Malik, S. A. et al. BH3 mimetics activate multiple pro-autophagic pathways. *Oncogene* **30**, 3918-3929 (2011).

7. Wu, L. et al. Specific small molecule inhibitors of Skp2-mediated p27 degradation. *Chem. Biol.* **19**, 1515-1524 (2012).
