# Supplementary material for: SKP2 attenuates autophagy through Beclin1-ubiquitination and its inhibition reduces MERS-Coronavirus infection
Source: Nat Commun. 2019 Dec 18;10:5770. doi: 10.1038/s41467-019-13659-4 (PMC6920372; doi:10.1038/s41467-019-13659-4)
Supplement: Supplementary file 3 — Reporting Summary [file 41467_2019_13659_MOESM3_ESM.pdf]

nature research

Corresponding author(s): Nils Gassen and Theo Rein

Reporting Summary

Nature Research wishes to improve the reproducibility of the work that we publish. This form provides structure for consistency and transparency in reporting. For further information on Nature Research policies, see Authors & Referees and the Editorial Policy Checklist.

Statistical parameters

When statistical analyses are reported, confirm that the following items are present in the relevant location (e.g. figure legend, table legend, main text, or Methods section).

n/a	Confirmed	
☐	☒	The exact sample size (*n*) for each experimental group/condition, given as a discrete number and unit of measurement
☐	☒	An indication of whether measurements were taken from distinct samples or whether the same sample was measured repeatedly
☐	☒	The statistical test(s) used AND whether they are one- or two-sided<br>*Only common tests should be described solely by name; describe more complex techniques in the Methods section.*
☐	☒	A description of all covariates tested
☐	☒	A description of any assumptions or corrections, such as tests of normality and adjustment for multiple comparisons
☐	☒	A full description of the statistics including central tendency (e.g. means) or other basic estimates (e.g. regression coefficient) AND variation (e.g. standard deviation) or associated estimates of uncertainty (e.g. confidence intervals)
☐	☒	For null hypothesis testing, the test statistic (e.g. *F*, *t*, *r*) with confidence intervals, effect sizes, degrees of freedom and *P* value noted<br>*Give P values as exact values whenever suitable.*
☒	☐	For Bayesian analysis, information on the choice of priors and Markov chain Monte Carlo settings
☒	☐	For hierarchical and complex designs, identification of the appropriate level for tests and full reporting of outcomes
☒	☐	Estimates of effect sizes (e.g. Cohen's *d*, Pearson's *r*), indicating how they were calculated
☐	☒	Clearly defined error bars<br>*State explicitly what error bars represent (e.g. SD, SE, CI)*

Our web collection on statistics for biologists may be useful.

Software and code

Policy information about availability of computer code

Data collection	No software was used
Data analysis	FACS data were analyzed with FlowJo software version 8.1. Statistical analyses were performed using the SPSS Statistics 18 software (IBM SPSS Statistics, IBM, Chicago, IL, USA).

For manuscripts utilizing custom algorithms or software that are central to the research but not yet described in published literature, software must be made available to editors/reviewers upon request. We strongly encourage code deposition in a community repository (e.g. GitHub). See the Nature Research guidelines for submitting code & software for further information.

Data

Policy information about availability of data

All manuscripts must include a data availability statement. This statement should provide the following information, where applicable:
- Accession codes, unique identifiers, or web links for publicly available datasets
- A list of figures that have associated raw data
- A description of any restrictions on data availability

The source data underlying Figures 1, 2, 4-9, and Supplementary Figures 1-10 are provided as a Source Data file. All other data is available from the authors upon request.

Field-specific reporting

Please select the best fit for your research. If you are not sure, read the appropriate sections before making your selection.

☒ Life sciences ☐ Behavioural & social sciences ☐ Ecological, evolutionary & environmental sciences

For a reference copy of the document with all sections, see nature.com/authors/policies/ReportingSummary-flat.pdf

Life sciences study design

All studies must disclose on these points even when the disclosure is negative.

Sample size	The experiments were performed in cell culture, thus typically analysing the average of millions of cells. This type of experiment was repeated at least three times, based on previous experience. When individual cells were analysed for counting autophagosomes and autolysosomes, at least 10 cells were analysed, again based on previous experience.
Data exclusions	No data were excluded.
Replication	The cell experiments were performed in at least three biological replicates.
Randomization	Not relevant for cell culture experiments
Blinding	The counting of autolysosomes and autophagosomes was performed by a colleague blind to the experimental conditions.

Reporting for specific materials, systems and methods

Materials & experimental systems

n/a	Involved in the study	
☒	☐	Unique biological materials
☐	☒	Antibodies
☐	☒	Eukaryotic cell lines
☒	☐	Palaeontology
☒	☐	Animals and other organisms
☒	☐	Human research participants

Methods

n/a	Involved in the study	
☒	☐	ChIP-seq
☐	☒	Flow cytometry
☒	☐	MRI-based neuroimaging

Antibodies

Antibodies used	Actin (1:5000, Santa Cruz Biotechnologies, sc-1616), Beclin-1 (1:1000, Cell Signaling Technology, #3738), K48-linkage Specific Polyubiquitin (D9D5) Rabbit mAb (Cell Signaling #8081, 1:500), PI3K3C (1:1000, Cell Signaling Technology, #4263), LC3-B (1:1000, Cell Signaling Technology, #3868), SQSTM1/p62 (1:1000, Cell Signaling Technology, #5114), VAMP8 (1:1000, Cell Signaling Technology, #13060), Atg14 (1:1000, Cell Signaling Technology, #5504), STX17 (1:1000, Sigma Aldrich, SAB001204), SNAP29 (1:1000, Sigma Aldrich, SAB2107406), PHLPP1 (1:1000, Millipore, 07-1341)
Validation	Actin (https://www.scbt.com/scbt/de/product/actin-antibody-i-19), Beclin-1 (https://www.cellsignal.com/products/primary-antibodies/beclin-1-antibody/3738), K48-linkage Specific Polyubiquitin (D9D5) Rabbit mAb (https://www.cellsignal.com/products/primary-antibodies/k48-linkage-specific-polyubiquitin-d9d5-rabbit-mab/8081), PI3K3C (https://www.cellsignal.com/products/primary-antibodies/pi3-kinase-class-iii-d9a5-rabbit-mab/4263), LC3-B (https://www.cellsignal.com/products/primary-antibodies/lc3b-d11-xp-rabbit-mab/3868), SQSTM1/p62 (https://www.cellsignal.com/products/primary-antibodies/sqstm1-p62-antibody/5114), VAMP8 (https://www.cellsignal.com/products/primary-antibodies/vamp8-antibody/13060), Atg14 (https://www.cellsignal.com/products/primary-antibodies/atg14-antibody/5504), STX17 (https://www.sigmaaldrich.com/catalog/product/sigma/sab1304559?lang=de&region=DE), SNAP29 (https://www.sigmaaldrich.com/catalog/product/sigma/sab1408650?lang=de&region=DE), PHLPP1 (http://www.merckmillipore.com/DE/de/product/Anti-PHLPP1-Antibody,MM_NF-07-1341)

Eukaryotic cell lines

Policy information about cell lines

Cell line source(s)	ATCC, DSMZ

Authentication	Upon purchase
Mycoplasma contamination	Checked by PCR-based method
Commonly misidentified lines (See ICLAC register)	*Name any commonly misidentified cell lines used in the study and provide a rationale for their use.*

Flow Cytometry

Plots

Confirm that:

☒ The axis labels state the marker and fluorochrome used (e.g. CD4-FITC).

☒ The axis scales are clearly visible. Include numbers along axes only for bottom left plot of group (a 'group' is an analysis of identical markers).

☒ All plots are contour plots with outliers or pseudocolor plots.

☒ A numerical value for number of cells or percentage (with statistics) is provided.

Methodology

Sample preparation	Cells were washed in PBS and fixed with cold ethanol (70%) for 30 min at 4 °C. After washing with PBS cells were treated with ribonuclease A (100 µg/ml; Sigma Aldrich) and stained with Vybrant DyeCycle Orange (Invitrogen, V35005) as described by the manufacturer's manual. Vybrant® DyeCycle™ Orange stain is excited using both 488 nm and 532 nm laser lines with emission ~570 nm.
Instrument	FACSCalibur (BD Biosciences).
Software	FlowJo software (Tree Star).
Cell population abundance	no sorting
Gating strategy	Gating corresponding to G1 or G2/M is indicated in the plots

☒ Tick this box to confirm that a figure exemplifying the gating strategy is provided in the Supplementary Information.
